# Self-Reported and Performance-Based Outcome Measures Estimation Using Wearables After Unilateral Total Knee Arthroplasty

**DOI:** 10.3389/fspor.2020.569932

**Published:** 2020-09-25

**Authors:** Ik-Hyun Youn, Todd Leutzinger, Jong-Hoon Youn, Joseph A. Zeni, Brian A. Knarr

**Affiliations:** ^1^Division of Navigation and Information Systems, Mokpo National Maritime University, Mokpo, South Korea; ^2^Department of Biomechanics, College of Education, University of Nebraska Omaha, Omaha, NE, United States; ^3^Department of Computer Science, College of Information Science and Technology, University of Nebraska Omaha, Omaha, NE, United States; ^4^Department of Rehabilitation and Movement Sciences, School of Health Professions, Rutgers University, Newark, NJ, United States

**Keywords:** KOOS, timed “up and go” test, self-report, accelerometer, wearable sensors, gait, total knee arthoplasty

## Abstract

Total knee arthroplasty is a common surgical treatment to improve ambulatory function for individuals with end-stage osteoarthritis of the knee. Functional and self-reported measures are widely used to assess functional ability and impairment before and after total knee arthroplasty. However, clinical assessments have limitations and often provide subjective and limited information. Seamless gait characteristic monitoring in the real-world condition is a viable alternative to address these limitations, but the effectiveness of using wearable sensors for knee treatment is unclear. The purpose of this study was to determine if inertial gait variables from wearable sensors effectively estimate the questionnaire, performance (6-min walk test, timed up and go, and 30-s chair stand test), and isometric measure outcomes in individuals after unilateral total knee arthroplasty. Eighteen subjects at least 6 months post-surgery participated in the experiment. In one session, three tasks, including self-reported surveys, functional testing, and isometric tests were conducted. In another session, the participants' gait patterns were measured during a 1-min walking test at their self-selected gait speed with two accelerometers worn above the lateral malleoli. Session order was inconsistent between subjects. Significant inertial gait variables were selected using stepwise regressions, and the contributions of different categories of inertial gait variables were examined using hierarchical regressions. Our results indicate inertial gait variables were significantly correlated with performance test and questionnaire outcomes but did not correlate well with isometric strength measures. The findings demonstrate that wearable sensor-based gait analysis may be able to help predict clinical measures in individuals after unilateral knee treatment.

## Introduction

Knee osteoarthritis (OA) is a common degenerative joint disease that decreases an individual's functional ability and overall quality of life (Ruiz et al., 2013; Palazzo et al., 2016). Total Knee Arthroplasty (TKA) is the most widely used surgical intervention for end-stage OA, and often results in improved knee joint function and quality of life (Palazzo et al., 2016). Given that the ability to regain ambulatory function following TKA is a major contributor to patient satisfaction and treatment success, the utilization of self-reported questionnaires and functional performance tests to monitor patient improvement can be useful clinical indicators to measure recovery (Curb et al., 2006; Lee et al., 2017). Studies investigating gait characteristics before and after TKA often use functional performance tests [timed up and go (TUG), 6-min walk test (6MWT), etc.] and self-report questionnaires to discriminate between individuals with proper and poor functioning, but these measures only provide a small snapshot of the individuals' functional abilities. Although questionnaires are cost-effective and easy to administer, they are subjective and may not accurately reflect physical impairments or functional deficits.

Previous research has clearly demonstrated that patient-reported outcomes alone do not accurately describe recovery post-TKA (Mizner et al., 2011), and that a combination of patient-reported and performance-based measures are needed to identify patients with functional deficits (Mizner et al., 2011; Bolink et al., 2015; Hossain et al., 2015). However, obtaining quantitative data for patients is time-consuming, may require access to expensive equipment, and is often unreasonable based on patient availability, especially in rural environments. Moreover, functional performance tests can objectively capture a patient's mobility, but each test only addresses a small aspect of physical function not fully capturing the subject's true experiences in everyday life. Given these challenges and short-comings, there has been great interest in using low-cost wearable sensors to develop mobile and remote tools for obtaining functional patient data. Previous studies exploring the use of sensor-based assessment post-TKA are limited to simple signal metrics such as spatiotemporal (Bolink et al., 2015) or peak acceleration measures (Christiansen et al., 2015), with no correlation to insightful biomechanical or performance based measures.

Wearable sensor-based mobility monitoring is a promising approach to overcome the limitations of common clinical measures (Chen et al., 2016). Wearable-based gait monitoring is low cost, portable, and can provide objective data of gait characteristics after unilateral TKA in a patient's natural environment (Komnik et al., 2015). In addition, the use of sensor based data can help determine if altered walking kinetics are related to poor clinical outcome measures, and if so give insight to how they are related. The use of wearable devices has been validated against self-reported questionnaire data in other chronic disease populations. For example, Rodríguez-Martín et al. (2017) used a triaxial accelerometer and machine learning to better detect freezing of gait in patients with Parkinson's disease as opposed to self-reported questionnaires, and Na and Buchanan (2020) validated the use of wearable technology against self-reported measures for stability in individuals with knee OA. Our previous work estimated kinematic and kinetic gait parameters from inertial gait variables using wearable devices (Youn et al., 2018), but the efficacy of inertial gait variables in determining self-reported patient outcomes and functional performance based measures in individuals post-TKA has yet to be explored. While it is often suggested that movement patterns are associated with functional ability and quality of life, few studies have evaluated the relationship between objective measures of gait and clinical outcomes.

Before wearable technology can be used to monitor and analyze patient gait characteristics, the effectiveness of the system needs to be carefully evaluated. Therefore, the purpose of this study was to determine if inertial gait features from wearable accelerometers were associated with self-reported and performance-based measures after unilateral TKA. Inertial gait parameters related to initial loading behavior (the initial 10% of the gait cycle) were selected since they are correlated with OA progression (Hatfield et al., 2015) This study is unique in the sense that it provides an original method for inertial gait variable extraction and estimation model development of clinical measures.

## Materials and Methods

**Figure 1:**
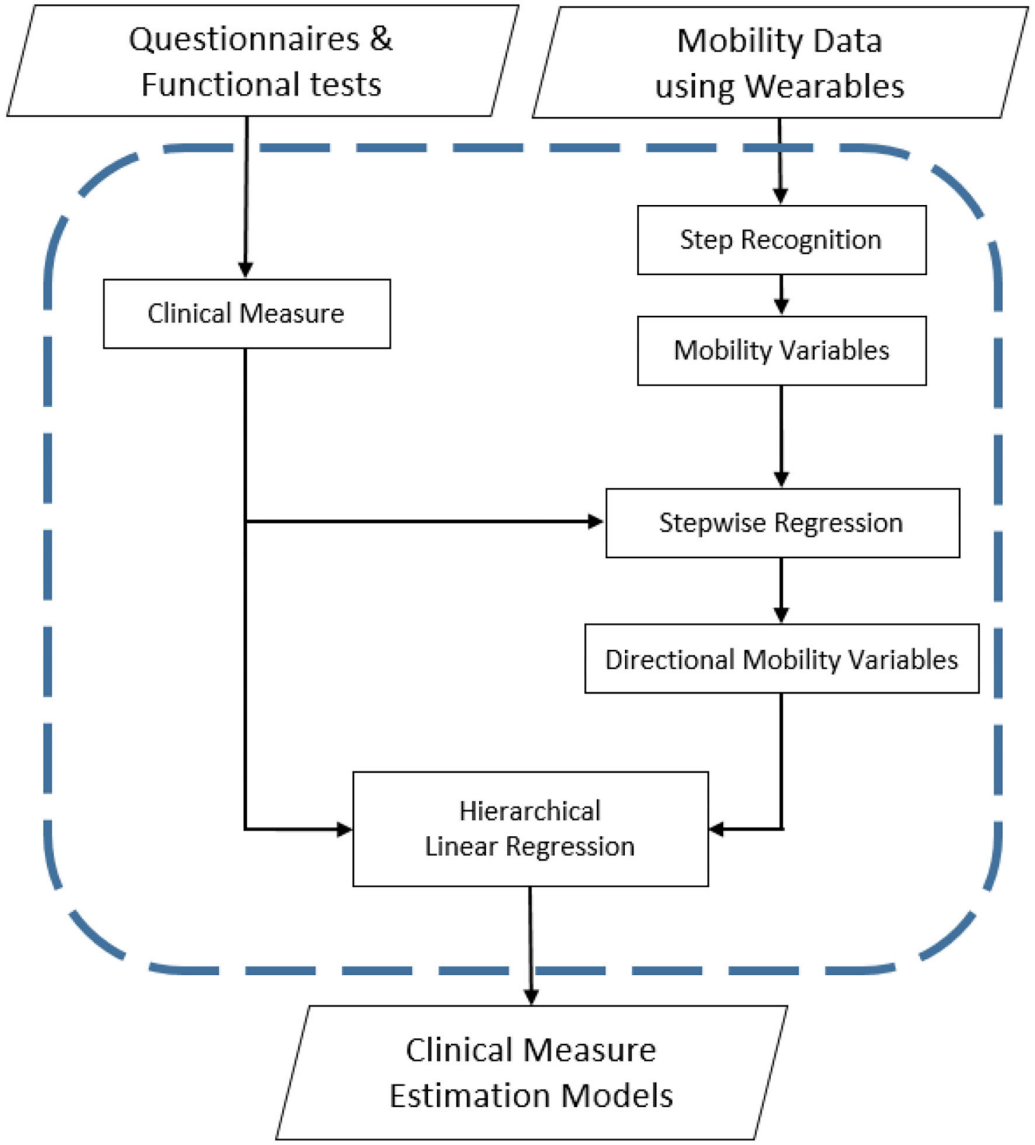
Step by step study methodology for developing clinical measure estimation models.

Figure 1 demonstrates each step of the research process for this study from data collection to estimation model development. During the collection of raw gait data from the wearable accelerometers, feature selection was performed to acquire statistically informative inertial gait variable subsets. The directional contribution of inertial gait variables to clinical measures was then determined through the creation of hierarchical linear regressions.

### Participants and Protocol

Data were acquired in the Neuromuscular Biomechanics Laboratory at the University of Delaware from 18 subjects (1.71 ± 0.08 m, 87.1 ± 17.5 kg, 66.5 ± 7.7 year, speed = 1.11 ± 0.19 m/s) at least 6 months post-unilateral TKA. To be included, subjects must have undergone total (not partial) knee arthroplasty at the local Center for Advanced Joint Replacement. All subjects followed a similar post-operative pathway that included several weeks of outpatient physical therapy after discharge from the hospital. Subjects were excluded from this study if they were unable to walk unassisted or had other neurological, cardiopulmonary, or orthopedic impairments that affected their ability to participate in functional testing. The University of Delaware Institutional Review Board approved the study, and each participant signed an informed consent form before commencing study protocol. Subjects performed a 1-min walk test on an instrumented split-belt treadmill (Bertec Corp, Columbus, OH, USA) at a self-selected walking speed. Self-selected walking speed was determined for each subject using a 6-m walk test. During the 1-min treadmill session, two ankle-worn accelerometers (Noraxon USA, Scottsdale, AZ) were attached to the subjects' legs above the lateral malleoli to collect three-dimensional acceleration data. Acceleration data were sampled at 200 Hz. For the sensor worn on the left leg, the X-axis pointed up to the shank, the Y-axis pointed backward, and the Z-axis pointed away to the left. The X-axis of the right sensor pointed up to the shank, the Y-axis pointed forward, and the Z-axis pointed outward to the right. The raw acceleration data were processed using custom software developed in the MATLAB 9.0 (Mathworks, Natick, MA) environment.

### Clinical Measures

Each participant answered self-reported surveys, performed functional testing, and completed isometric strength testing. The self-reported surveys included the Knee Outcome Survey Activities of Daily Living Scale (KOS-ADLS) and the Knee Injury and Osteoarthritis Outcomes Score (KOOS) which consists of three subscales, KOOS-Pain, KOOS-Symptom, and KOOS-Quality of Life (KOOS-QOL). The functional testing performed by each subject included the 6MWT, TUG, and 30-s chair stand test (30 s-CST). Isometric strength testing was performed only with the surgical limb using a Kin-Com (Chattecx Corp., Hixson, Tennessee) isometric dynamometer. Subjects were seated in the device with their surgical limb flexed and stabilized at 60 degrees. Subjects were instructed to kick out with their leg as hard as they could. Strength was quantified by the amount of torque produced by the subject. This was performed at least two times and the maximum was used in data analyses. Verbal encouragement was provided.

### Gait Analysis Using Wearable Sensors

#### Normalization

Previous research has demonstrated that normalization of joint torques is highly effective in reducing individual differences among participant data (Moisio et al., 2003). To account for the confounding effect of participant anthropometric differences, the raw acceleration data were normalized by height.

#### Inertial Gait Variable Extraction

Once normalizing raw acceleration data, the step recognition algorithm applied. As the anterior directional gait motion of the lower limbs was greater compared to the medial and vertical directions, gait event recognition was performed using the anterior directional acceleration. At each heel-strike a large peak was generated in the anterior directional acceleration indicating initial loading within a gait cycle. The step recognition method was validated previously in a mobility analysis study (Youn et al., 2016, 2017), and was applied to each sensor, respectively. Following the completion of individual step recognition, peaks from the two accelerometers and raw acceleration data were merged to generate data of each subject's step cycles.

Eleven gait variables were extracted from the three-dimensional accelerometer data to estimate the magnitude, impulse, and angles of initial loading for each subject. These sensor-based gait variables were then compared to the questionnaire and performance test outcomes. Hierarchical linear regressions were applied to determine the contribution of directional gait variables from wearables for each clinical measure. To analyze the directional effect of inertial gait variables, anterior, vertical, lateral, and inclusive-gait inertial variables were sequentially added to prior regression models. Gait variables were extracted from the initial 10% of stance phase, the initial 10% of the directional impulse, and the maximum directional acceleration at heel strike. Furthermore, whole step vector magnitude (i.e., the length of the 3-D vector), ankle angle variation (i.e., standard deviation of angle) in lateral and anterior directions, and step time were calculated to describe whole step characteristics (Table 1).

**Table 1:** Inertial gait variable properties.

**Purpose**	**Acronym**	**Description**	**Method**
Step magnitude initial step magnitude	VM	Whole step vector magnitude	Vector magnitude of the whole step
	VM10	Initial 10% of step vector magnitude	Initial 10% of whole step vector magnitude
Directional magnitude of initial loading	MAG-L	Lateral heel-strike magnitude	Maximum lateral acceleration at HS
	MAG-V	Vertical heel-strike magnitude	Maximum vertical acceleration at HS
	MAG-A	Anterior heel-strike magnitude	Maximum anterior acceleration at HS
Directional impulse of initial loading	IMP-L	Lateral heel-strike impulse	Initial 10% SD of lateral acceleration
	IMP-V	Vertical heel-strike impulse	Initial 10% SD of vertical acceleration
	IMP-A	Anterior heel-strike impulse	Initial 10% SD of anterior acceleration
Directional ankle angle variation during stance phase	ANG-L	Lateral stance phase angle variation	SD of lateral stance phase angle
	ANG-A	Anterior stance phase angle variation	SD of anterior stance phase angle
Temporal parameter	ST	Step time	HS-to-HS time

### Data Analysis

A Pearson's correlation analysis was used to quantify the relationship between all independent (i.e., 11 inertial gait variables) and dependent variables (i.e., eight clinical measures). The 11 inertial gait variables were categorized by direction, i.e., lateral, anterior, vertical, or inclusive (overall magnitude and step time) for all statistical analyses.

To reduce the amount of mutual information and avoid overfitting the estimation models, stepwise regression analysis was used to analytically select the most relevant inertial gait variables for the eight clinical measures (Grossman et al., 1996; Boulet et al., 2016). An increase in the adjusted *R*^2^-value of the stepwise regression was required for variable inclusion in the model. *K*-fold cross-validation (Milner, 2008; Gunaratne et al., 2017) was applied with *k* = 10 to improve model robustness. In *k*-fold cross-validation, all study subjects (*n* = 18) were randomly partitioned into ten subgroups. Nine subgroups were used as training data for the model, while the remaining subgroup was used for validation and testing the model. The cross-validation process was repeated ten times (*k* = 10), with each subgroup being used exactly once as the validation data. The procedure was performed with the intent to make the estimation models more robust to account for variations in TKA patients gait data compared to healthy controls and to improve the overall validity of model predictions.

Hierarchical linear regressions were used to determine which directional inertial variables had the greatest predictive power on the clinical measure estimation models through stepwise addition of selected inertial variables from each directional category into the model (Masse et al., 2016). Separate regressions were conducted for each of the eight clinical measures. For example, to establish the TUG estimation model, anterior variables were entered into the model first, followed by the vertical and lateral inertial variables, respectively. For all hierarchical regressions, the inclusive inertial variables were entered at the last step. Model *R*^2^ and the significant change in *R*^2^ between each step were evaluated. A significant increase in model *R*^2^ was used to determine if the inertial variable provided increased predictive power.

## Results

Overall, inertial variables were significantly correlated with self-reported survey and performance test results (Table 2). Impulse and magnitude inertial variables including vertical heel-strike magnitude and vertical heel-strike impulse were significantly correlated with self-reported survey and performance tests (Table 3).

**Table 2:** Mean and standard deviation of clinical measures.

**Clinical Measures**	**Mean ± Standard deviation**
KOS activities of daily living scale	87.45 ± 7.91
KOOS pain	86.88 ± 12.31
KOOS symptom	76.25 ± 17.53
KOOS quality of life	79.84 ± 14.13
6MWT	561.66 ± 105.63
TUG (s)	6.86 ± 1.41
30 s CST (repetitions)	16.28 ± 4.34
Average quadriceps torque (Nm)	165.81 ± 45.81

KOS = Knee Outcome Survey.

KOOS = Knee Injury and Osteoarthritis Outcomes Score.

6MWT = six-min walk test.

TUG = timed up and go test.

*30 s CST = 30 s chair stand test*.

**Table 3:** Selected inertial variables using stepwise regression to predict clinical measures.

**Category**	**Clinical measures**	**Description**			
		**Lateral**	**Vertical**	**Anterior**	**Inclusive**
Self-reported survey	KOS-ADLS	MAG-L	MAG-V	ANG-A	ST
	KOOS-Pain	-	IMP-V	-	ST VM
	KOOS-Symptom	-	-	MAG-A ANG-A	VM10
	KOOS-QOL	ANG-L	IMP-V	MAG-A ANG-A	VM
Functional testing	6MWT	MAG-L ANG-L	-		ST
	TUG	MAG-L	IMP-V	-	VM ST
	30 s-CST	ANG-L	IMP-V	MAG-A	VM ST
Isometric tests	Torque	-	-	-	VM ST

Based on the criteria of stepwise regression (i.e., an increase in adjusted *R*^2^-value), independent inertial gait variables for predicting each clinical measure were selected (Table 2). All directional and inclusive gait variables were selected for KOS-ADLS, KOOS-QOL, and 30 s-CST test, whereas only one-directional gait variable was selected for the isometric test estimation model (i.e., anterior heel-strike magnitude only for strength test). The selected gait variables are expected to reduce mutual information between the 11 gait variables with smaller subset sizes.

The gait variables selected using stepwise regression were applied to the hierarchical linear regression. Overall, hierarchical linear regression results demonstrated a strong potential that the proposed wearable sensor-oriented acceleration data could assist in quantifying clinical gait measures. Results of the hierarchical linear regression indicated that inertial gait features were significantly related to self-reported measures (Table 4), and performance tests, but the isometric strength tests did not demonstrate a meaningful relationship to the inertial gait features (Table 4). All models performed well at explaining the variance in self-reported measures. Gait variables explained 89.3, 54.6, 70.3, and 63% of the variance in the KOS-ADLS, KOOS-Pain, KOOS-Symptom, and of KOOS-QOL scores, respectively.

**Table 4:** Hierarchical linear regressions result for self-reported survey estimation.

**Self-reported**												
**Hierarchy**	**KOS-ADLS**			**KOOS-pain**			**KOOS-symptom**			**KOOS-QOL**		
	***R***	**Adj**. ***R***^**2**^	**Δ** **R**^**2**^	***R***	**Adj**. ***R***^**2**^	**Δ** ***R***^**2**^	***R***	**Adj**. ***R***^**2**^	**Δ** ***R***^**2**^	***R***	**Adj**. ***R***^**2**^	**Δ** ***R***^**2**^
Anterior	0.28	0.23	0.23	0	0	0	0.67	0.64	0.64	0.3	0.25	0.25
Vertical	0.43	0.28	0.05	0.38	0.34	0.34	0.67	0.64	0	0.34	0.23	−0.02
Lateral	0.48	0.27	−0	0.38	0.34	0	0.7	0.65	0	0.5	0.36	0.13
Inclusive	0.94	0.89	0.62	0.64	0.55	0.21	0.76	0.7	0.05	0.77	0.63	0.27
**Functional**												
**Hierarchy**	**6MWT**			**TUG**			**30 s-CST**			**Torque**		
	***R***	**Adj**. ***R***^**2**^	**Δ** ***R***^**2**^	***R***	**Adj**. ***R***^**2**^	**Δ** ***R***^**2**^	***R***	**Adj**. ***R***^**2**^	**Δ** ***R***^**2**^	***R***	**Adj**. ***R***^**2**^	**Δ** ***R***^**2**^
Anterior	0	0	0	0	0	0	0.36	0.25	0.25	0	0	0
Vertical	0	0	0	0.21	0.08	0.08	0.4	0.24	−0.01	0	0	0
Lateral	0.44	0.4	0.4	0.21	0.08	0	0.53	0.35	0.11	0	0	0
Inclusive	0.77	0.71	0.31	0.75	0.68	0.6	0.8	0.68	0.33	0.28	0.23	0.23

Functional tests were explained well by combinations of the four directional categories. For the 6MWT 71.2% of the variance was explained by lateral and inclusive gait variables, while 68.2% and 68.4% of the variance in the TUG and 30 s-CST test, respectively were explained by the selected gait variables. In contrast, the isometric tests did not present a meaningful relationship. Only 23.3% of the variance in knee extension torque was explained by anterior gait variables.

Regarding overall clinical measure relationship results, seven self-reported and functional test results were significantly related by using selected subsets of inertial gait variables. Additionally, directional contributions were identified. For instance, the KOS-ADLS result was primarily related to the anterior axis and inclusive gait variables, and the inclusive gait variables predicted most of the clinical measures (i.e., 0.618 of 0.893 as adj. *R*^2^). Similar directional alignments were observed from KOS-QOL. Although TUG results were significantly related to the inertial gait varabiables, there was no such directional agreement because none of the anterior, vertical, or lateral gait variables improved the results.

## Discussion

The goal of this research study was to determine if inertial gait variables from wearable sensors could be used to effectively estimate self-reported, functional performance, and isometric strength outcomes in individuals post-unilateral TKA. Overall, we found that the proposed novel method of extracting clinical measures from 3D accelerations is capable of showing a significant relationship between key clinical measures in a post-unilateral TKA population. What was particularly striking was the strength of the associations, which ranged as high as explaining 89% of the variation in clinical outcome. This suggests that movement patterns, in particular inertial measurements from the lower limb, are highly related to performance. While this has been suggested to be the case in numerous other studies, the strong relationship provides a rehabilitation for clinicians who aim to improve functional outcomes with movement retraining. It also suggests that this technology may have substantial benefit in monitoring patient progress for those who do not have the ability to attend in-person rehabilitation or follow-up clinical sessions.

Previous literature have used other measures to show a relationship between functional and self-reported outcomes post-TKA, but our results demonstrate greater correlations between variables (Curb et al., 2006; Ruiz et al., 2013; Palazzo et al., 2016; Lee et al., 2017). Mizner et al. (2005) investigated quadriceps strength (*R*^2^ ≤ 0.54) to predict TUG and KOS scores and Stevens-Lapsley et al. (2010) investigated body mass index (BMI) (*R*^2^ ≤ 0.63) to predict TUG, 6MWT, and KOS scores. These results indicate wearable sensor data are more highly correlated to clinical measures and questionnaires compared to previous methods used to assess health and overall function in individuals post-TKA. This increased relationship may largely be due to the direct nature of wearable accelerometry during gait, compared to indirect measures of body stature (BMI), pre-disposition (limb alignment), or capacity (strength) that do not take into account an individual's active movement and muscle coordination during the specific task of gait.

Outcomes of the regression models indicated that inertial gait features were significantly related to self-reported measures and performance tests but were not closely related to isometric strength. Of the 11 inertial variables, ten were significantly correlated with one or more clinical measure. Both lateral heel-strike magnitude and anterior stance phase angle variation were not significantly correlated with any of the selected clinical measures, while the KOS-ADLS was significantly correlated with eight inertial variables. Previous research relating self reported measures to sensor based gait parameters have shown mixed results. For example one study found no significant relationship between West Ontario McMaster Universities Osteoarthritis Index and spatiotemporal gait parameters taken from inertial gait sensors in individuals post-total hip arthroplasty (Bolink et al., 2016). However, these results were from simple spatiotemporal parameters and did not measure acceleration data as done in the current study. Another study focused on TKA rehabilitation found high correlation between sensor based outcome measures and questionnaire data for some subjects and not others (Calliess et al., 2014). Youn et al. (2018) estimated joint kinematics and kinetics from acceleration data collected using two ankle-worn sensors and found that step time was the only inertial gait variable not significantly correlated with knee flexion moment, knee adduction moment, anterior ground reaction forces, or vertical ground reaction forces, but both lateral heel-strike magnitude and anterior stance phase angle variation were significantly correlated with one or more gait variables (Youn et al., 2018). Differences in these results may be due to the difference in estimated measures between the two studies (biomechanical gait parameters vs. functional performance tests and questionnaires) despite both using wearable sensors to investigate a post-unilateral TKA population.

The primary axes of each self-reported measure, clinical test, and isometric strength test were calculated through hierarchical linear regressions to determine the directional contributions of the inertial variables to clinical measures (Masse et al., 2016). Interestingly, anterior inertial variables were not used in the prediction of both the 6MWT test and TUG but were used in prediction of 30 s-CST. Lateral and inclusive variables were most predictive of 6MWT test and all three variables except those in the anterior direction were used to predict TUG. This is not immediately intuitive given the significance of propulsion and forward gait speed in 6MWT and TUG performance but could suggest the importance of lateral stability and balance in performance-based measures (Podsiadlo and Richardson, 1991; Harada et al., 1999; Shubert et al., 2006). During the 30 s-CST the subject does not move anteriorly but simply attempts to stand up and sit down in a chair as many times as possible in 30 s. Unfortunately, the complexity of gait makes it difficult to explain connections between the inertial variables and self-reported measures of this study, however, by using inertial gait variables to predict patient outcomes as opposed to functional tests, we are able to achieve insight that is not intuitive and would not be obtained from the clinical measures alone. An example of this is previous study that was able to report improvements in patient-reported outcome measures post-TKA, despite no global gait parameter differences before and after surgery (Kluge et al., 2018). This result is in line with previous research which has found increases in patient reported outcome measures despite no improvements in walking function (Calliess et al., 2014; Bolink et al., 2015). However, with the use of pre-operative sensor-based gait parameters, the authors were able to predict which subjects would improve post-surgery with up to 89% accuracy, something that cannot be achieved by questionnaires or functional tests alone.

It is logical that surveys investigating everyday life such as the KOS-ADLS and the KOOS-QOL were accurately predicted by inertial gait variables in all four directions, because movement in everyday life is not one dimensional, but requires motion in a variety of different directions. Moreover, previous literature has shown a lack of strong correlation between lower limb strength and walking speed (Bohannon et al., 1996; Bohannon, 1997). This lack of a strong relationship between these two variables compliments our finding that inertial gait variables did not predict isometric strength scores well. Walking at a self-selected speed is not a maximal strength task, and therefore would not be likely to provide relevant information on maximal strength. Maximum walking speed has been shown to be better correlated with lower extremity strength measures compared to self-selected walking speed and may provide better insight on maximal strength measures when using inertial gait variables (Bohannon et al., 1996; Bohannon, 1997).

By demonstrating inertial gait variables are effective in estimating self-report questionnaires and functional performance tests wearable-based gait monitoring can guide clinical decision-making by addressing questions that are important but currently difficult to answer in a clinical setting. These include: “Is the current physical therapy program having an effect on performance outside of the clinic?,” “Is the patient following a normal trajectory of function?,” and “Does the patient have persistent asymmetries that are not being resolved with the current plan of care?” Based on the answers to these questions, a clinician can use this information to address deficiencies in a patient's rehabilitation program.

This study represents the first step in a line of research exploring the utility of inertial measures in this patient population. The clear link between movement patterns and clinical outcomes suggest that inertial measurements may serve as a target for rehabilitation, or as a surrogate measure of clinical outcomes in patients who need remote monitoring. Future research should determine if the relationship present in the current study changes with kinetic strength testing or fast walking speeds as opposed to the isometric strength testing and self-selected walking speeds performed in this study.

## Conclusions

The proposed models and clinical measure estimation results provided evidence that inertial measurements can be used to estimate standard clinical measures. Although cross-validation was applied, the generalizability of these results to the entire TKA population could be limited due to a small study sample size. Future work will examine the relationship between fast walking speeds and their ability to predict clinical outcomes from ankle-worn accelerometer data.

## Data Availability Statement

The raw data supporting the conclusions of this article will be made available by the authors, without undue reservation.

## Ethics Statement

The studies involving human participants were reviewed and approved by University of Delaware Institutional Review Board. The patients/participants provided their written informed consent to participate in this study.

## Author Contributions

I-HY and J-HY conceived and designed the paper. JZ and BK designed the experiments. JZ, BK, and TL contributed to data analysis and interpretation. All authors contributed to writing and reviewing the paper.

## Conflict of Interest

The authors declare that the research was conducted in the absence of any commercial or financial relationships that could be construed as a potential conflict of interest.
